# Mechanistic Basis of Nanobubble Functionality in Food Systems: Linking Electrokinetic Stabilization, Transport Dynamics, and Functional Outcomes

**DOI:** 10.1111/1541-4337.70602

**Published:** 2026-08-22

**Authors:** Farah Naqash, Miral Javed, Akmal Nazir, Sajid Maqsood

**Affiliations:** ^1^ Department of Food Science, College of Agriculture and Veterinary Medicine United Arab Emirates University Al Ain UAE; ^2^ Department of Integrative Agriculture, College of Agriculture and Veterinary Medicine United Arab Emirates University Al Ain UAE; ^3^ Sustainable Food Engineering Lab College of Engineering and Physical Sciences University of Guelph Guelph Ontario Canada; ^4^ ASPIRE Research Institute for Food Security in the Drylands (ARIFSID) United Arab Emirates University Al Ain UAE

**Keywords:** electrokinetic stabilization, matrix modulation, microbial modulation, nanobubbles, transport persistence

## Abstract

Recently, there has been a surge in shifting of scientific interest towards the adoption of nonthermal technologies in food processing applications. Nanobubble (NB) technology is one of the emerging green technologies that has gained increased attention over recent years. However, its functionality is often discussed separately from the gas identity, electrokinetic stabilization, transport behavior, and food matrix interactions. This review develops a framework based on mechanism–property–transport–function–outcome to explain how NB stability, interfacial charge, residence time, sustained transport, and matrix interactions govern food functionality and applications. This framework explains why similar NBs perform differently across food systems, emphasizing and classifying dominant mechanisms of NB action for food applications, along with gas properties as an underlying factor. This review bases electrokinetic and transport phenomena as mechanistic foundations of the stability and applicability of NBs in food systems, while highlighting gas‐specific, property‐tuned, and food‐matrix‐directed framework for selecting NBs for potential food applications. The outcome of electrokinetic and transport‐based interactions with the food matrices has been discussed under residence time‐governed transport, micro‐convection effects, food matrix modulation, redox reactions, and bioactive delivery.

## 1 Introduction

Nanobubble (NB) technology has rapidly gained attention as a versatile and innovative approach to addressing critical challenges in science. It is demonstrating significant potential across a broad spectrum of applications, including environmental remediation, enhancement of industrial process efficiencies, improvements in food and agricultural systems, advancements in personal care formulations, and the development of novel diagnostic and therapeutic strategies in biomedical fields. Being a green technology coupled with exceptional stability and functional efficiency, it is attracting attention in food processing as well. NBs are nanoscopic, usually gaseous cavities in liquid solutions capable of changing the characteristics of water (Kyzas et al. 2019). Two types of NBs that have been principally studied include bulk and surface NBs, with the former at the solid–liquid interface and the latter in the bulk liquid, typically with a diameter ranging from 100 to 200 nm and stable for extended periods. Surface NBs are generally pocket‐filled gas with a height of about 10 nm and an area of 7850–78,500 nm^2^. Bubbles less than 1 µm in size and suspended in liquid are referred to as bulk NBs (Kyzas et al. 2019). Due to their size in the nano range, they exhibit distinct structural properties that differ from those of macro‐bubbles with ordinary attributes. Some of the structural attributes of NBs as studied by atomic force microscopy have identified the radius of curvature of NBs of the order of 100 nm, height to be 20–30 nm, and contact angle to be between 5° and 20° (Walczyk et al. 2013). Surface NBs are generally stable for up to 4 days owing to their small size despite having a smaller contact angle. Surface NBs exist in a dynamic equilibrium as the outflux of gas from the interface is balanced by influx on the interface (Brenner and Lohse 2008). Apart from NBs at the surface, bulk NBs have been studied extensively over the last few years. One of the intrinsic characteristics of bulk NBs is their stability, which ultimately dictates their applications in various realms, and is in turn defined by surface charge intensity and electrostatic interaction among two surfaces. The exceptional stability of bulk NBs often is a point of debate and needs an extensive understanding for potential applications, calling for molecular computational approaches for exploring their interactions in bulk and at the interface (Michailidi et al. 2020). The longevity and extended stability of NBs, usually ranging from several weeks to months, are a paradox in itself as it contradicts classical thermodynamic theories. As per Young–Laplace law, the pressure internal to the bubble is inversely proportional to its diameter, and therefore, small bubbles owing to a high internal pressure should dissolve immediately, but that not being the case makes these NBs exceptional in applications (Kyzas et al. 2019). Unique properties, including stability, free radical formation, negative zeta potential, surface attraction, and oxidation, confer these NBs with the added advantage of being able to control pathogens, prevent mineral scaling, biofilm formation, and improve solid/oil/liquid separation (Y. Zhou, Han, et al. 2021). NBs are generally regarded as nonthermal at bulk processing level as the technology does not involve application of heat; however, localized thermal effects are observed in systems based on cavitation (Takahashi et al. 2007). NBs come to the rescue of processes facing lacunae in mass transfer from gas to liquid streams, since decreasing bubble size reduces the rising velocity of bubbles and increases the surface area to volume ratio. As the rising velocity of NBs is lower than their Brownian motion velocity, they find applications in bioprocess enhancement. NBs stay in the liquid for a considerable time and expose a large interfacial surface area as opposed to the same volume of bubbles in the macroscopic range. The superiority of NBs over other gas‐transfer methods is also rooted in the fact that these remain suspended in the liquid and, unlike other bubbles, do not disappear or coalesce, consequently upkeeping continuous gas transfer until final collapse (Patel et al. 2021). However, existing literature discussing the NBs does not link mechanistic behavior of the NBs with their functionality in food systems. Therefore, in this review, the interplay of thermodynamic, kinetic, and interfacial effects that enable both the stability and functionality of NBs has been explained as a property arising out of these interactions, that later decide the specific food functionality outcome. Existing reviews have mainly discussed NBs’ generation, stability, electrokinetic behavior, mass transfer, and food applications as separate themes (Phan and Bhandari 2025; C. Xu et al. 2025). This separation does not explain why the same NBs produce contrasting outcomes in food systems, such as promoting desirable microbial growth in fermentation while inactivating pathogens during sanitation. Food functionality ultimately emerges from the coupling and interaction between electrokinetic stabilization, transport behavior, and matrix‐mediated interfacial interactions. An explanation of efficacy and varying functionality stemming from enhanced gas exchange, hydrodynamic interactions, redox reactions, and gas functionality at the nanoscale needs to be put through, to better understand their functionality in a specific food system or matrix. Therefore, this review uses electrokinetic stabilization and transport behavior as the mechanistic foundation but extends them into a property–function–outcome framework governed by gas identity, redox behavior, matrix interactions, and intended application. This framework introduces novel insights, and it emphasizes that the stability governed by electrokinetics, will not alone produce functionality or the transport persistence alone is not sufficient for desired outcomes; therefore, different manifestations of this coupling produce different food functionalities. This approach is crucial for providing a basis for selecting NB systems according to processing, preservation, microbial, and quality‐control objectives.

## 2 Origin and Electrokinetic Basis of NBs

### 2.1 Nucleation and Interfacial Stabilization

**FIGURE 1:**
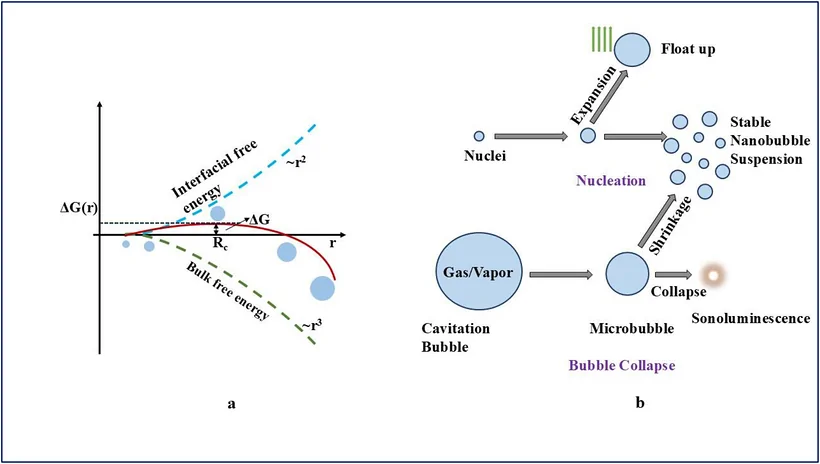
Principles and mechanism for bulk nano‐bubble generation: (a) classical nucleation theory for bubble formation and (b) two possible pathways leading to the formation of stable bulk nano‐bubble suspensions. Redrawn and modified from L. Zhou, Wang et al. (2021) with reproducing permission from Elsevier under license number 6104141496486.

The formation of bulk NBs involves nucleation of dissolved gas followed by the stabilization of newly formed gas–liquid interface. Nucleation is a first‐order phase transition reaction that involves gas emerging from a liquid that is in a metastable state (Figure 1) (L. Zhou, Wang, et al. 2021). If there are no pre‐existing bubbles in the solution, homogeneous nucleation takes place. However, it is difficult to achieve because an energy barrier exists for bubbles to overcome, and consequently the free energy change is represented as

(1) $$\Delta G=\frac{4}{3}\;\pi R^{3}G_{v}+4\pi R^{2}\gamma$$

where ΔG represents the energy loss or gain for system to allow bubble formation with radius *R*, $G_{v}$ being the energy associated with unit volume of gas (it is negative and hence promotes nucleation). γ, the liquid–vapour surface tension, acts against nucleation as it has a positive value. When the gas is supersaturated, a decrease in free energy barrier is seen consequently favoring bubble formation (Weijs et al. 2012). On the other hand, nucleation that is heterogeneous is attributed to the presence of impurities that reduce the surface area of nuclei, thereby lowering surface free energy. Therefore, for generating bulk NBs, heterogeneous nucleation in environments with an excess of gas is a prerequisite (L. Zhou, Wang, et al. 2021). Additionally, surfactants and hydrophobic molecules are seen to reduce surface tension and modify charge distribution, which favors the formation and stabilization of NBs (Ma et al. 2022). These interfacial phenomena are important as they establish the electrokinetic characteristics that define NB behavior. Surface charge and interfacial stabilization are particularly important as these contribute towards NB persistence, which further influences the transport behavior and functionality in food systems.

### 2.2 Bubble Growth Dynamics

From the nucleation of a bubble to it reaching the final size, diffusive growth governs the growing pattern of bubbles. The bubble growth with time is related by a square root power law equation as under

(2) $$R\left(t\right)∼{\left(Dt\right)}^{1/2}$$

where *D* is the diffusion constant of the gas, *R* is the radius, and *t* is the time. The dissolved gas concentration outside a bubble in a liquid body is given by Henry's law as

(3) $$\frac{p}{\phi }=k_{H}$$

where *p* represents the partial pressure of the gas, ϕ is the concentration of the specific gas in the liquid, $k_{H}$ is the Henry's constant. With decrease in bubble size, the Laplace pressure and interfacial tension are seen to influence gas dissolution. However, the liquid layer between neighboring bubbles alters the local concentration gradient and restricts gas diffusion from the bubble, as a result of which NBs exhibit prolonged existence irrespective of nanoscale dimensions (Weijs et al. 2012). The persistence of such concentration gradients can be of importance in liquid food systems as it influences parameters like electrokinetic stabilization, gas retention, and transport‐mediated interactions that involve food components and reactive species. The growth dynamics, therefore, establish basis for electrokinetic stabilization that is a prerequisite for any food‐related functionality.

## 3 Generation Methods of NBs

The generation method of NBs varies depending on the type desired. Commonly reported methods for surface NBs are based on solvent exchange, electrochemical reactions, and immersing hydrophobic substrate into water with altering pressure and temperature (Foudas et al. 2023). However, as bulk NBs draw more attention among the scientific community, their generation finds relatively greater mention in the scientific literature. Generally, bulk NBs are produced by mechanical stirring, pressure fluctuation, gas dissolution release, and cavitation, as well as by microfluidic membrane methods. Development and selection of an efficient, cost‐effective, scalable, and reliable method are key to the adoption of this technology at the commercial and industrial level. The various methods of bulk NB generation are discussed in the following sections:

### 3.1 Cavitation

Cavitation‐based methods are widely adopted approaches for the generation of bulk NBs as they are known to produce intense activity at the interface and enhanced transport behavior. Cavitation comes into effect when the static pressure of a liquid drops below vapor pressure, giving rise to small cavities filled with vapor within the liquid. When these cavities burst, it generates turbulence, shear forces, shock waves, and interfacial disturbances that contribute to new bubble formation as well as subsequent transport amplification. Along with these mechanical effects, collapse is accompanied by generation of localized hot spots. The local hot spots are confined to a nanoscale, have lifetimes of few microseconds, and do not cause a measurable rise in food temperature; however, local effects can cause protein denaturation, nutrient loss, enzyme inactivation, and DNA disruption through generated radicals and shear forces depending upon intensity, exposure, and food matrix (X. Sun et al. 2021). Cavitation mechanisms for formation of bulk NBs are principally identifiable as vaporous and gaseous. In the event of pressure level falling below the vapor pressure of liquid, there is rapid transformation of liquid into vapor, leading to bubble formation. This is known as vaporous cavitation and is generally recognized as acoustic cavitation, mostly caused by sound. However, if the pressure falls below the saturation pressure of gas in the liquid, a diffusion process comes into effect and is known as gaseous cavitation generally recognized by the term hydrodynamic cavitation (Mallesham et al. 2025).

#### 3.1.1 Hydrodynamic Cavitation

**FIGURE 2:**
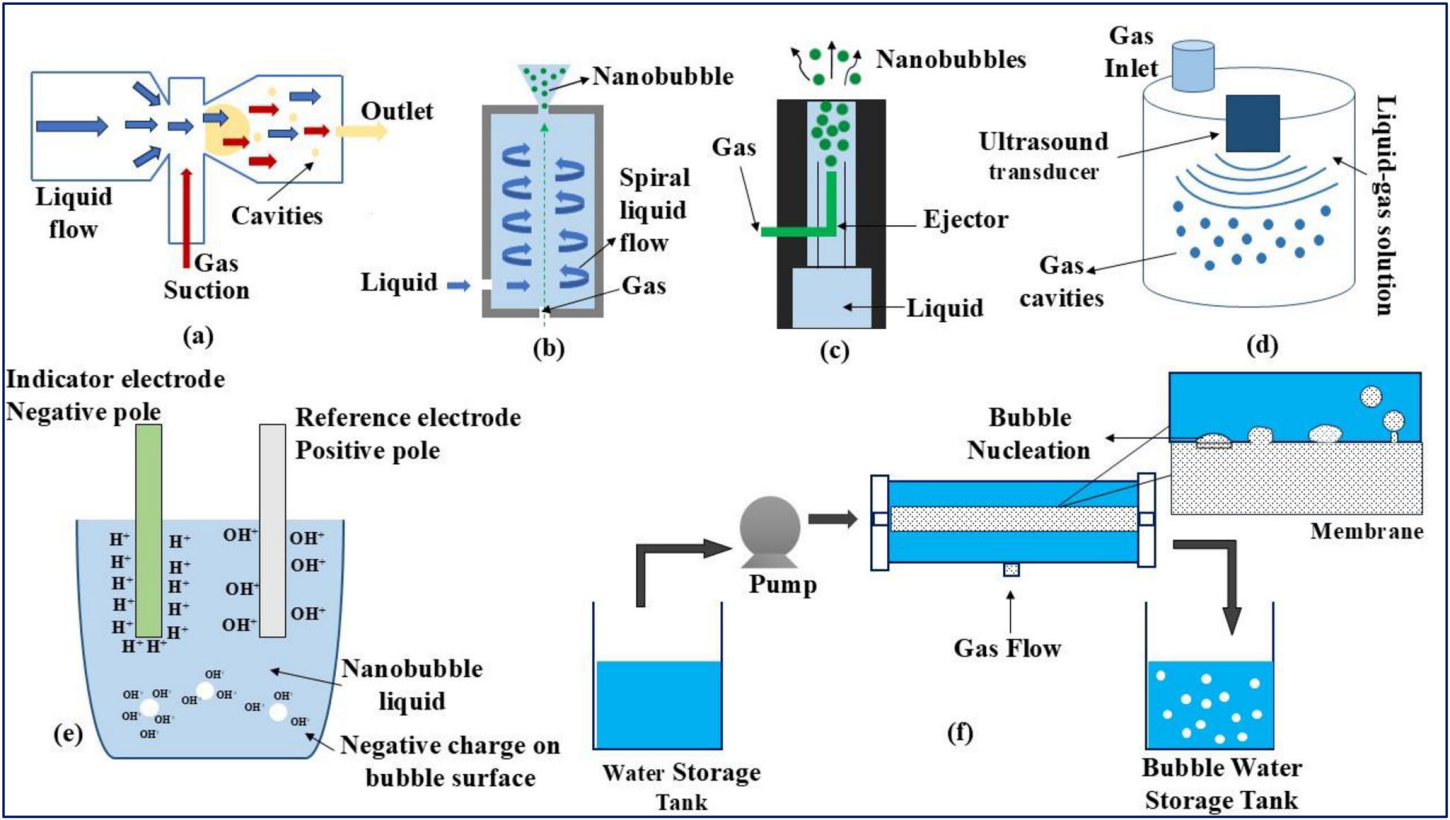
Schematic diagram of different techniques used to generate NBs—(a) Venturi system; (b) spiral liquid flow; (c) ejector type system of hydrodynamic cavitation; (d) ultrasonic system; (e) electrolysis method; and (f) membrane system. Redrawn and modified from Mallesham et al. (2025) and T. Phan et al. (2020) with reproducing permission from Elsevier under license number 6280070495768 and 6280070176108.

Hydrodynamic cavitation occurs when a flowing liquid experiences a sudden pressure reduction owing to changes change in shape and structure of the system. It is characterized by the occurrence of nucleation followed by bubble expansion and finally bubble implosion. Hydrodynamic cavitation is considered one of the most energy‐efficient and economically feasible methods for large‐scale NB production. The construction of generation devices is divided into two categories, one with mobile parts such as that of a rotor–stator assembly and another with nonmoving parts, which include linear flow meters such as orifice and venturi (Figure 2a), and swirling flow devices (Simpson and Ranade 2019). Variations in pressure and flow velocity regulate NB size and concentration within these systems (T. Phan et al. 2020). In a spiral liquid flow (Figure 2b), a liquid is pumped into a cylindrical chamber through a side orifice, causing a swirl to flow that further forms a vortex. When the gas is introduced into the spiral liquid from the bottom, NBs are formed as a result of centrifugal force of the rotating liquid (Khuntia et al. 2012). Similarly, ejector‐based systems (Figure 2c) generate NBs by ejection of gas into regions of rapid expansion with lowest pressure (Mallesham et al. 2025).

Hydrodynamic cavitation is particularly relevant in food‐processing systems because the generated NBs exhibit strong collapse‐induced transport amplification, turbulence, and increased diffusion (Boczkaj et al. 2023). Such characteristics favor applications involving extraction of bioactive compounds, membrane cleaning, antifouling, and intensified mass transfer. However, the loophole associated with this otherwise effective and affordable technology is the generation of heat that can possibly interfere with the NB attributes and consequently their stability (Akshit et al. 2024).

#### 3.1.2 Acoustic Cavitation

Acoustic cavitation comes into effect when a liquid is subjected to ultrasound waves with a frequency above 20 kHz and enough intensity to create bubbles from existing gas nuclei (Figure 2d). Oscillating pressure fields generated by ultrasound promote repeated compression and rarefaction cycles, resulting in nucleation, expansion, and collapse of gas nuclei within the liquid medium (Babu and Amamcharla 2022). The collapse of cavitation bubbles generates intense shear forces and shock waves that enhance mass transfer, disrupt cell membranes, and facilitate the mixing and dispersion of solutes. Consequently, the NBs generated are characterized by strong diffusional amplification, enhanced solvent penetration, and increased interfacial transport. These characteristics render acoustic cavitation suitable for extraction systems, microbial disruption, and transport‐intensified food‐processing applications. Influence of ultrasonic power, exposure time, and frequency is seen on the diameter and concentration of NBs, establishing these conditions to be critical for the NB generation process (Bu and Alheshibri 2021). Ultrasonic cavitation is relatively advantageous over other methods due to operation and ability to vary the parameters for controlling concentration and size of NBs (M. Li et al. 2021).

### 3.2 Electrolysis

The electrolytic method involves the usage of an electrode with positive and negative poles as sites of NB generation (Figure 2e). It is distinct from other methods, as gas NBs are formed as a result of electrolysis of various salts at the electrode (Babu and Amamcharla 2023b). Electrolysis mediated by the electrode causes decomposition of water, thereby generating hydrogen and oxygen gases, ultimately leading to formation of stable oxygen and hydrogen NBs. When the concentration of these gases touches level of supersaturation at the anodic and cathodic ends of liquid, NBs are formed (Zhu et al. 2016). Oxygen NBs have a relatively larger lifetime than that of hydrogen NBs, the latter having a life of a few hours. In the initial stages, the gas bubbles tend to nucleate and grow on the electrode, then detach and float due to buoyancy and interfacial tension (L. Zhou, Wang et al. 2021). NBs generated by this method have a high interfacial charge and therefore more pronounced stabilization, which favors sustained residence and reactive species generation (Yadav et al. 2024). These systems can, therefore, be applied for oxygen‐based decontamination and reactive oxygen species (ROS) transport. Electrolytic method is advantageous for its scalability, energy efficiency, and cost‐effectiveness. Increasing the applied current, operating time, and electrolyte concentration and reducing water temperature is seen to multiply the generation of NBs (Mallesham et al. 2025; Ranaweera and Luo 2020). Owing to its scalability, cost‐effectiveness, and ability to generate highly charged NBs, electrolysis remains a promising approach to be explored for food applications.

### 3.3 Membrane Method

In membrane‐based method, a gaseous phase is steered into an aqueous phase through a nano‐porous membrane, generating NBs at the interface of the two phases. As the air passes through a thin, permeable layer, a negative pressure is induced leading to the formation of bubbles (Figure 2f) (Mallesham et al. 2025). The formation mechanism is primarily based on the expansion of bubbles followed by their detachment. It is the surface tension between the water and air that holds NBs near the pore, whereas the drag force of aqueous phase pulls them apart, therefore causing their detachment from the pore (Kukizaki and Goto 2006). Compared with cavitation‐based methods, membrane systems produce NBs with narrower size distributions and improved colloidal stability (Ansari et al. 2025). Consequently, membrane‐generated NBs can be suitable for applications requiring sustained gas transfer, prolonged transport persistence, and controlled oxygenation behavior. For the generation of gaseous NBs, the application of ceramic membranes for protein dispersion systems has proven to be better suited than hydrodynamic cavitation, in that the latter causes foaming and hence reduces process efficacy. Moreover, the cavitation set up is complicated, underscoring the need for continuous membrane‐based generation of NBs (Babu and Amamcharla 2023b; Dhungana and Bhandari 2021). The bubble characteristics, such as size and shape, are influenced by pore diameter of the membrane. A generalized setup of membrane‐based NB generator consists of a pressurized gas tank, gas flow meter, gas pressure regulator, and porous membrane (Mallesham et al. 2025).

### 3.4 Ethanol–Water Exchange

The solvent exchange process or the ethanol–water exchange, works by displacement of a solution saturated with a gas or oil (ethanol) with a solvent, like water, creating oversaturation for nucleation. This method is mostly applicable for the fabrication of surface NBs; however, bulk NBs are also formed by solvent‐exchange (Qiu et al. 2017). Some studies claim the entities from solvent exchange to be nanodroplets or nanoparticles, whereas others hold the formation to be actual gas NBs. The former opinion finds foundation on the response of nanodrops to external pressure and their density, whereas the latter examines solvent exchange process to identify a decrease in the size of bubbles during exchange, atypical to gas NBs (Alheshibri and Craig 2019; M. Chen et al. 2020). Compared with cavitation and membrane methods, solvent exchange approaches presently exhibit limited applicability in food‐related bulk NB systems owing to toxicity constraints and scalability issues.

### 3.5 Miscellaneous Methods

Other methods of NB generation include the decompression‐type and gas–water circulation type in which incorporation of gas in the continuous liquid is increased at high pressure, leading to supersaturation. This is followed by decompressing the solution to atmospheric pressure creating NBs (Ikeura et al. 2011). NBs have also been fabricated by depressurized gas‐saturated solution through a needle valve, by the usage of electric fields in gas–liquid systems as well as by vibration (Calgaroto et al. 2014; Fang et al. 2020; Ghaani et al. 2020).

## 4 Gas‐Dependent NB Behavior and Food Functionality

The gas identity forms an important defining factor for interpreting functionality of NBs in food systems as it dictates the stability, mass transfer, interfacial activity, and redox reactions based on distinct chemical and physical interrelated effects. The reader is referred to the review of Wang et al. (2026) for a detailed account of gas composition and applications. It is a general observation from studies that the stability of NBs generated from various gases is highest for oxygen and ozone, followed by air and nitrogen, and the least for carbon dioxide, principally attributable to solubility differences and zeta potential (T. Phan et al. 2020). Stable O_2_ NBs are seen to favor sustained release of the gas, such that O_2_ NBs can promote prolonged availability of oxygen for aerobic metabolism, fermentation, probiotic growth, and bioprocesses limited by oxygen but can also promote oxidative disinfection if linked with reactive species (Shi et al. 2026; Silva et al. 2024; Ulatowski and Sobieszuk 2020). On the other hand, N_2_ NBs are based on non‐oxidative preservation principally by displacing dissolved oxygen, thereby reducing oxidation–reduction potential and therefore providing an inert atmosphere for food preservation (K. Zhang, Feng, et al. 2026). CO_2_ NBs show a transient behavior involving bursting followed by dissolution as they are highly soluble and therefore less stable, leading to the release of dissolved CO_2_, lowering pH and supporting acidification‐based preservation in liquid or high‐moisture foods (T. Phan et al. 2020). O_3_ NBs exhibit oxidative characteristics, acting as advanced oxidation systems at the interface by decomposition into •OH radicals that causes microbial inactivation, degradation of pesticide/organic contaminants, and biofilm disruption (Pal and Kioka 2025). However, H_2_ NBs act as antioxidative or redox‐protective entities than disinfectants, because hydrogen NBs selectively scavenge ROS such as •OH, ClO^−^, and ONOO^−^ and show higher antioxidant activity than hydrogen water without NBs owing to its lower water solubility (Liu et al. 2018). Therefore, H_2_ NBs should be differentiated from O_3_ NBs that cause microbial inactivation by promoting oxidative stress for microbial inactivation, whereas H_2_ NBs reduce the oxidative stress through selective ROS scavenging.

In addition to gas identity, bubble size also affects NB behavior and should be given due consideration for designing food applications. Ultrasmall NBs (<50 nm) have been shown to inhibit oxidation caused by reactive radicals, whereas normal NBs show a weak, however, favorable oxidation behavior dependent on the substrate. Ultrasmall NBs selectively quench unstable ROS at the gas–liquid interface, whereas larger NBs may provide interfacial area that is sufficient to adsorb both ROS and substrates, thereby increasing the possibility of their reaction. The antioxidant effect of ultrasmall NBs is relatively sustainable than conventional reducing agents as the gas–water interface is not depleted. This also distinguishes antioxidative effect based on size from H_2_ NBs, where antioxidant activity is linked to the chemistry of hydrogen gas and persistence of NB implying that the redox behavior of NBs depends on gas size distribution along with gas type (Zheng et al. 2023). This antioxidative behavior can be applied for foods liable to oxidation, where ultrasmall NBs may help to delay lipid oxidation, prevent pigment degradation, and loss of sensitive bioactive compounds without the need for adding exogenous antioxidants. In fresh‐cut fruits and vegetables, ultrasmall NBs could be explored as a strategy to reduce browning caused by ROS as well as oxidative stress; however, experimental validation is required in real food systems. For foods rich is lipid, such as meat, fish, dairy, and edible oils, the ability of ultrasmall NBs to scavenge ROS can suggest limiting oxidative rancidity along with preserving color, flavor, and nutritional quality. Ultrasmall NBs can act as antioxidants in bioactive delivery systems and may protect nutraceuticals that are sensitive to oxidation, synergizing to the benefit of encapsulation matrices.

The methods of generation also have an impact on NB properties that can later define the functionality. During generation of N_2_ NBs, parameters like water pressure, gas flow, and shearing time were seen to impact regulated bubble density, zeta potential, ORP, surface tension, and viscosity, indicating that food applications require generation of NBs in a manner that tailors their properties compared to generalized usage. N_2_ NBs also showed higher stability than O_2_ and CO_2_ NBs, as they retained their density considerably after 30 days and maintained partial stability even under conditions of high‐salt, which can be favorable for preservation of foods high in salt (K. Zhang, Feng, et al. 2026). The inherent nature of NB also dictates their stability and functionality. It was evident from CO_2_ NBs of 3, 5, 7, and 10 nm, where the stability was seen to increase with size. Larger CO_2_ NBs were characterized by a multilayer structure, and comprised of a dense core, transition, and exchange layer, and exhibiting lower diffusion and gas exchange rates, whereas smaller NBs lacked a stable core and showed greater water penetration, consequently dissolving more readily. The internal multilayer conferred stability to CO_2_ NBs in the absence of ions/surfactants and without shielding effects as well (R. Zhang, Yin, et al. 2026).

These properties highlight the variation in gas functionality and can enable the selective application of NBs in food systems depending whether the target process requires gas transfer, oxygen exclusion, oxidative inactivation, acidification, or antioxidative protection, and a careful selection can therefore enable process efficiency.

## 5 Electrokinetically Regulated Transport Mechanisms Governing Food Functionality

### 5.1 Electric Double Layer and Ionic Strength‐Mediated Transport Behavior

The stability of NBs fundamentally governs their sustained transport, interfacial reactions, mass transfer ability and consequent functionality within food systems. It has been established that the stable nature of NBs contradicts the classical phenomenon in physics as per the Young–Laplace equation (Equation 4), which postulates the shrinkage and consequent disappearance of small NBs owing to a high internal pressure:

(4) $$P=P_{0}+\frac{2\gamma }{R}$$

where *P* is the NB internal pressure, *P*_0_ is the pressure external to the NB, *γ* is the surface tension, and *R* is the radius. Moreover, as predicted by Epstein–Plesset theory and classical diffusion theory, for NBs with a diameter of less than 1 µm, the lifetime should be 0.05 s (Epstein and Plesset 1950). However, stable persistence of NBs over extended timespans ranging from hours to months indicates that additional stabilization mechanisms govern their interfacial and transport behavior (Foudas et al. 2023; Sun et al. 2022; L. Zhou, Wang et al. 2021). The electrostatic stabilization arising from interfacial charge has gained increased attention as it directly dictates the transport process. In aqueous systems, the charge of NBs is greatly defined by H^+^ and OH^−^ ions, and NBs exhibit a dominant negative charge at the interface (Calgaroto et al. 2014), which is in turn the result of excessive hydration of hydrogen ions such that the hydroxyl ions are rendered open to interact with the gas phase (Gurung et al. 2016). Accumulation of ions at the interface forms an electrical double layer (EDL), which suppresses coalescence and retards gas diffusion, thereby prolonging NB persistence (Ushikubo et al. 2010; Babu and Amamcharla 2023b). As the NB surface is negatively charged, the ionic strength and the gas solubility at interface are modified by potential at the double layer (Nirmalkar et al. 2018). The EDL repels the similarly charged ions and attracts counter‐ions leading to change in local viscosity and diffusivity (Firouzi et al. 2015). Therefore, electrokinetic interactions not only influence the NB stability, but also define the transport activities at interface that ultimately governs sustained gas diffusion within food systems.

**FIGURE 3:**
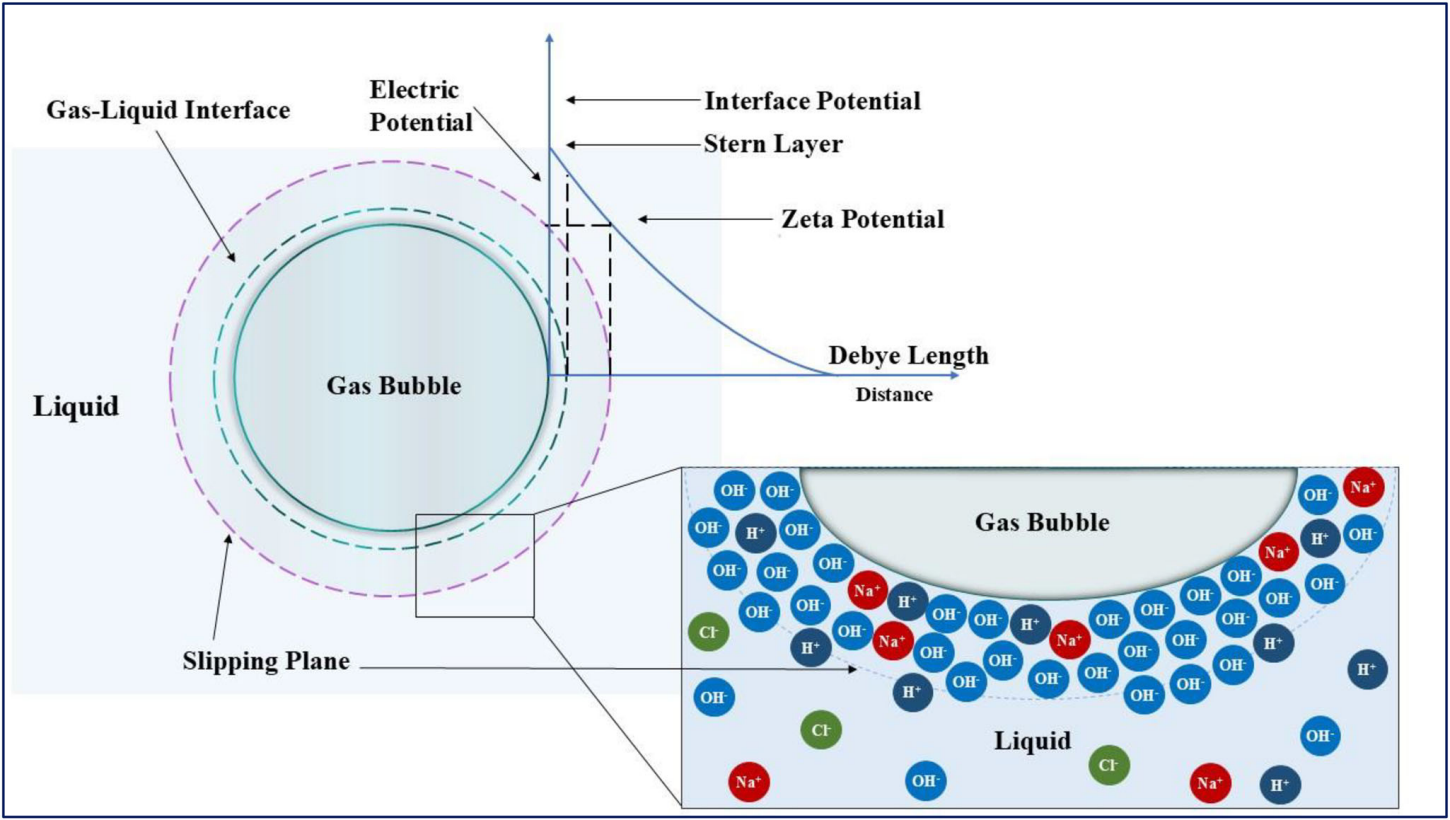
Schematic representation of a typical nano bubble in bulk liquid phase with the electric and potential layers (www.moleaer.com).

At the slipping plane or boundary layer (Figure 3), which divides the layer formed between counter ions on bubble interphase and bulk solution (T. Phan et al. 2020), a higher zeta potential confers higher stability to the NBs. A low absolute zeta potential is not able to prevent the aggregation and coalescence of NBs due to van der Waals forces (Haris et al. 2020). Lower surrounding pH values decrease the absolute zeta potential and also mobility of NBs, whereas a higher pH of the solution confers higher zeta potential to the NBs, due to anion (OH^−^) adsorption on surface of NBs thereby preventing their coalescence and prolonging NB persistence (Khodadadi Darban and Ahmadi 2013; Meegoda et al. 2018). As food systems exhibit substantial variations in pH and ionic composition, matrix conditions can strongly regulate NB transport behavior and gas retention functionality.

Ions adsorbing at the boundary of gas–liquid promote mutual ionic repulsion and a consequent reduced internal pressure. In a solution, a total negative charge prevalent on the surface of NB exerts a radial negative electrostatic pressure, balancing the internal Laplace pressure to confer stability to the NB (Ma et al. 2022). The surface charge density σ and the radius of the NB are inversely related as

(5) $$R=\frac{\gamma \varepsilon }{\pi \sigma^{2}}$$

Moreover, surface charge density and surface potential (*ψ*_0_) are linked by the Grahame equation as

(6) $$\sigma =\sqrt{8k_{B}T\varepsilon \varepsilon_{0}C_{\infty }}\sin h\left(\frac{ze\psi_{0}}{2k_{B}T}\right)$$

where σ is the surface charge density, $k_{B}$ represents Boltzmann constant, *T* is temperature, $\varepsilon \varepsilon_{0}$ is the dispersed medium and vacuum permittivity, $C_{\infty }$ is the co‐ion concentration in bulk solution, *z* is the salt valence, *e* is the elementary charge, and $\psi_{0}$ the surface potential. Reduction in surface potential decreases electrostatic pressure and accelerates gas dissolution, thereby destabilizing NBs under acidic conditions (Nirmalkar et al. 2018).

The colloidal stability of bulk NB clusters is explained by Derjaguin, Landau, Verwey, and Overbeek (DLVO) theory (Bunkin 1992) which explains stability as a balance between electrostatic repulsion and van der Waals attraction, as per Equation (5).

(7) $$\omega_{T}\left(D\right)=\omega_{R}\left(D\right)+\omega_{A}\left(D\right)$$

where $\omega_{T}$, $\omega_{R}$, and $\omega_{A}$, represent the total interaction potential, electrostatic potential, and van der Waals potential. A total interaction potential that is positive creates an energy barrier that prevents NB coalescence, usually above pH 4. Under reduced surface charge or high electrolytes, the electron double layer can be compressed or screened, thereby lowering electrostatic repulsion (Nirmalkar et al. 2018). Counter‐ions and salts modify interfacial properties and hydrodynamic boundary layers, thereby influencing gas retention and transport persistence (Firouzi et al. 2015).

The EDL thickness is characterized by the Debye length (Debye length = 1/κ):

(8) $$\kappa =\sqrt{\frac{2nz^{2}e^{2}}{\varepsilon_{r}\varepsilon_{0}kT}}$$

where κ is the Debye–Huckel parameter, *n* refers to the anion or cation concentration in solution, *z* is the ion valency, *e* is the electron charge, $\varepsilon_{r}$ is the relative dielectric constant, and $\varepsilon_{0}\;\mathrm{is}$ the vacuum permittivity. Therefore, NBs with thicker electric double layer and higher zeta potential will exhibit greater colloidal stability and a prolonged transport persistence (Y. Zhou, Han, et al. 2021).

These electrokinetic effects hold importance in food systems due to food composition such as proteins, polysaccharides, salts, minerals, and acids that can alter the ionic strength and consequently interfacial charge distribution. The ionic strength further influences EDL thickness and therefore NB behavior. In food systems like dairy products, juices, and protein solutions, the dissolved salts may compress the electric double layer and reduce Debye length, decreasing zeta potential and consequently favoring NB dissolution. Reduced electrostatic stabilization may shorten the residence time and limit sustained gas transfer. However, a lower ionic strength and higher negative zeta potential favor prolonged NB stability leading to sustained persistence of gases such as oxygen and ozone and their consequent transfer (Hewage et al. 2021; Y. Zhou, Han, et al. 2021). Similarly, proteins and polysaccharides can modify the surface charge and electric double layer on adsorption. Proteins can induce destabilization by such charge neutralization (Witkowski et al. 2025), whereas negatively charged polysaccharides can confer charge enrichment and stabilization (Zhang et al. 2020). As sustained transport of gases depends upon interfacial stability, the food matrix interactions ultimately regulate oxygen retention, ROS persistence, and gas‐transfer functionality within food systems. The various mechanistic applications of NBs in food systems are listed in Table 1.

**TABLE 1:** Applications of nano bubbles in the food and allied domains.

Application	Process parameters	Mechanism	Outcome	References
Freezing and crystallization	CO_2_ and N_2_ bubbles, low power ultrasound 205 kHz	Act as sites for nucleation	Lactose crystallization	Adhikari et al. (2018)
Protein purification	CO_2_ nano bubbles in an apolar–polar system passed through a sintered glass disk with flow rate of 3 L/min	Foam fractionation	Enhanced purity of protein hydrophobin	Khalesi et al. (2016)
Seed sprouting	Air, CO_2_, and N_2_ nano bubbles under 404 kPa and 0.45 L/m flow rate	Generation of reactive oxygen species promoting cell wall loosening and cell elongation	Increase in germination rate of barley seeds	Ahmed et al. (2018)
Defouling	Bathing pool assembly, electrochemical method of generation, H_2_ nano bubbles for 15 cycles	Free radical generation and bubble collapse	Removal of bovine serum albumin from stainless steel	Chen (2009)
	Air NBs, flow rate of 5.83–7.50 × 10^−6^ m^3^/s		Removal of humic acid from ceramic membrane	Ghadimkhani et al. (2016)
Extraction of bioactives	Rhamnolipid was used, extraction time (16 min)	CO_2_ NBs, rhamnolipid coated the NBs, reduced the surface tension which acted as nano jets	Increased the extraction of polyphenols from *Camellia oleifera* shells	Javed, Belwal, Ruyuan, et al. (2022)
	CO_2_ NBs, optimized parameters were ultrasonic power 358.76 W, surfactant concentration 4.54%, and time 41.41 min	CO_2_‐NBs acted as nano jets on the surface of peanut shells	Enhanced the phenolic compounds extraction from peanut shells, CO_2_‐NBs assisted ultrasonic technique achieved the highest antioxidant activity	Iqbal et al. (2025)
	N_2_ NBs, ionic liquid concentration of 1 M, ultrasonic power 350 W, extraction time 9 min, 30°C temp, and sample to solvent ratio of 0.5 g/10 mL	N_2_ NBs acted as nano jets	Increased the phenolic extraction from walnut shells	Ettoumi et al. (2022)
	CO_2_ NBs, power 240 W and 20 kHz frequency with a pulse mode of 1 s ON and 1 s OFF. Temp 40°C, time 16 min	CO_2_‐nano jets	Extraction efficiency of phenolic compounds from *C. oleifera* shells was increased	Javed, Belwal, Huang, et al. (2022)
	N_2_ NBs, power 250 W, 10°C, and 40 min	Disruption of cell walls by nano‐jets	Enhanced terpene compounds release, solubility, and overall yield	Sheikh et al. (2025)
Dairy products	Air NBs, A venturi injector with flow rate of 7.57 L/min	Disruption of milk protein aggregates by nano bubbles	Reduced the viscosity of milk protein concentrate	Babu and Amamcharla (2023a)
	Air NBs, A venturi injector with air flow rate (0.03 L/min)	Followed the hydrodynamic cavitation, and disrupted the large aggregates	Improved the rheological, microstructural and functional properties of Greek style yogurt	Babu et al. (2022)
Viscosity reduction	CO_2_ NBs, membrane method of generation, 300 kPa gas pressure for 5, 13, and 26 min of circulation time	Act as buffers among dispersed particles and prevent their aggregation	Reduced viscosity in apple juice concentrate and canola oil	Phan et al. (2021)

### 5.2 Residence Time‐Governed Gas Transport Functionality

The transport‐based functionality of NBs is fundamentally governed by their prolonged residence time and sustained gas‐transfer capability. Conventionally, gas transfer from NBs is based on gas diffusion driven by Laplace pressure; however, the electrokinetic stabilization significantly modulates the transport behavior around NBs (Nirmalkar et al. 2018). The surface charge of the electric double layer stabilizes NBs and influences sustained gas transfer. Therefore, a correction factor may be incorporated into the mass‐transfer equation as

(9) $$\frac{dC}{dt}=k_{L\alpha }\left(C_{s}-C_{\infty }\right)f\left(\zeta ,I,r\right)$$

where $\frac{dC}{dt}$ is the volumetric gas‐transfer rate, $k_{L\alpha }$ is the volumetric mass transfer coefficient, $C_{s}$ and $C_{\infty }$ represent gas concentration at the interface and in the bulk liquid, respectively, whereas f(ζ,I,r) represents the electrokinetic modulation and depends on zeta potential, ionic strength, and radius. When f(ζ)>1 electrokinetic stabilization prolongs NB lifetime and sustains the interfacial area available for diffusion (Nirmalkar et al. 2018).

**FIGURE 4:**
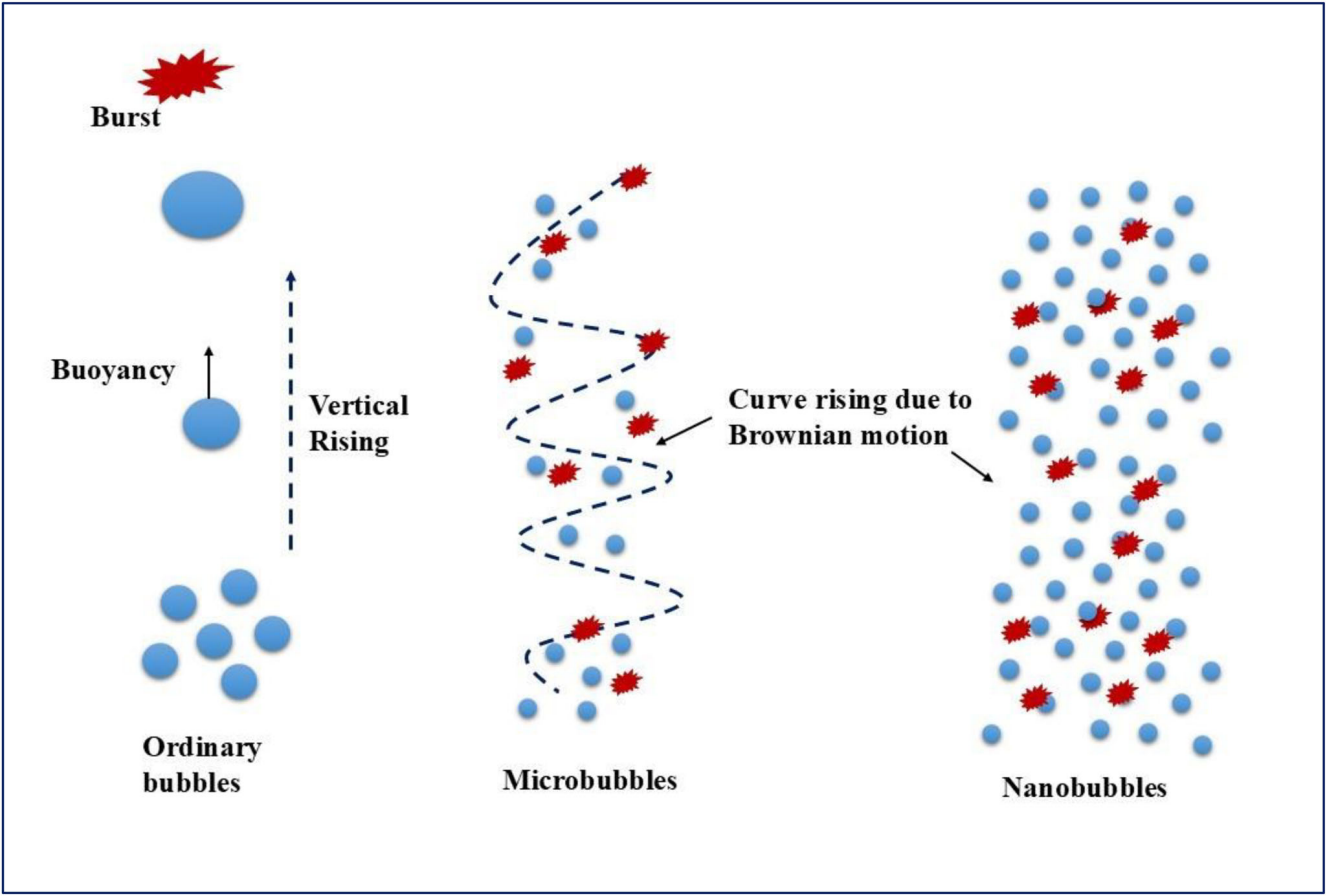
Comparative interpretation of rising process of ordinary bubbles and micro‐nano‐bubbles. Redrawn and modified from Zhang et al. (2023) with reproducing permission from Taylor and Francis under license number 6091360741029.

**FIGURE 5:**
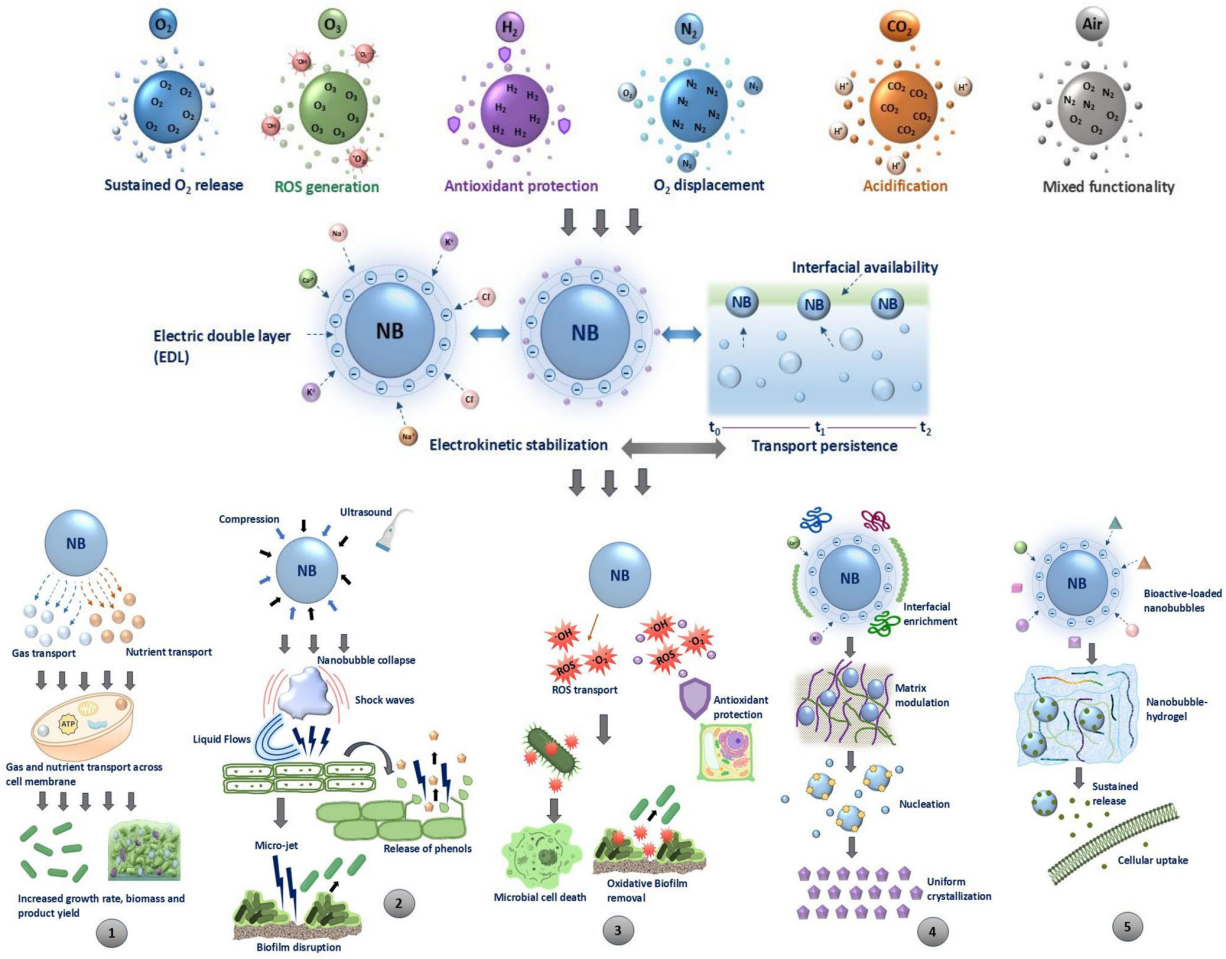
Mechanistic basis of nano‐bubble functionality in food systems: Electrokinetic stabilization and transport persistence based (1) residence‐time‐governed transport, (2) interfacial micro‐convection, (3) redox functionality, (4) matrix modulation, and (5) bioactive delivery.

The volumetric mass‐transfer coefficient (*k_L_* α) is, therefore, influenced by NB residence time:

(10) $$k_{L}\alpha \propto A/Vf\left(\tau_{bubble}\right)$$

where *A*/*V* is the interfacial area per unit volume and *τ*_bubble_ is the lifetime of the NB extended by the electrokinetic stabilization (H. Sharma et al. 2024). NB also exhibits long residence times in the bulk liquid, supported by a slower rising velocity to the surface as a result of lower buoyancy compared to larger bubbles (Z. H. Zhang et al. 2023). The motion of NBs is also seen to be affected by their colliding with water molecules in the near vicinity, creating Brownian motion, which prevents their rising to the surface, retains irregular curved motion and hence a high residence time in solutions (Babu and Amamcharla 2023a) (Figure 4) a high residence time favors effective interaction with food components, microorganisms, and surfaces (Silva et al. 2024) (Figure 5). Additionally, the NBs also possess a large specific surface area, which is the surface area per unit of volume and is inversely proportional to the diameter. Because of this, NBs possess a higher point of contact with substances, increasing the reaction of molecules with that of NBs in bulk liquid (Agarwal et al. 2011). The high surface area also renders more active sites needed for chemical reactions, adsorption processes, and interactions with biological or environmental targets. Owing to the larger specific area of NBs, the gas content dissolving into the liquid phase through the interface would increase with increasing pressure in and around bubbles. This promotes a higher gas‐transfer efficiency of NBs in the bulk (Agarwal et al. 2011). This is because small bubbles have a higher surface tension and rising internal pressure ultimately leading to collapse. Although shrinking, as the NBs undergo self‐pressurization, efficiency of gas transfer is enhanced at the gas–liquid interface. After supersaturation of gas in liquid, the gas transfer still continues, and this improves solubility of air, carbon dioxide, oxygen, ozone, and other gases in solution (Temesgen et al. 2017). These gas transport characteristics are consequently seen to directly influence microbial and biochemical processes within food systems.

The gas transport brought about by NBs has been interpreted as a strategy to intensify various bioprocesses, where the core gas and mode of supply of NB determine microbial growth and metabolite production. In a study carried by Guo et al. (2019), the microbial effects of NBs were strongly related to density of NBs, zeta potential, pH, and growth phase. N_2_ and H_2_ NBs produced strongest growth‐promoting effects followed by air, whereas CO_2_ showed little or no marked effect. N_2_ NBs exhibited highest density and strong negative zeta potential that reduced the lag phase and increased maximum growth rate, followed by H_2_ in a dose‐dependent manner. Stable NBs may act as carriers for ions or nutrients assisting in their transport towards bacterial cells and help the cells to adapt in early phase of growth. In the later stages, accumulation of lactic acid causes a decrease in pH and consequently reduces NB stability, arresting the growth‐promoting effect in stationary phase. CO_2_ NBs, however, reduce NB stability owing to low pH and weakest negative zeta potential, consequently limiting their possible biological effect (Guo et al. 2019). Similarly, oxygen NBs were seen to stimulate the aerobic growth of *Saccharomyces cerevisiae*, however not as a sole source for oxygen. Addition of oxygen NBs to aerated cultures produced higher biomass, rapid glucose consumption, and increased specific growth rate. The NBs were proposed to create local supersaturation zones near bubble interfaces, improving oxygen bioavailability thereby stimulating cellular metabolism, however under continued supply (Sobieszuk et al. 2021). NBs form an alternative route for substrate transfer into bacteria in addition to traditional diffusion supporting their growth (Guo et al. 2019). Similarly, controlled oxygen transfer mediated by NBs has been shown to improve oxygen utilization and sustain aerobic metabolism (Yaparatne et al. 2022). Sustained oxygen transport can minimize oxidative stress and also suppress the formation of undesirable metabolites, including aldehydes and organic acids (Silva et al. 2024). This can be instrumental in ensuring desirable flavor and stability of fermented products. Moreover, as substrate utilization is enhanced due to a sustained gas supply, it can increase the process efficiency along with less production of waste. For gas‐based mass transfer in bioreactors, gas transfer takes place from bubble to liquid phase to be consumed by the microorganisms later. Increasing the surface area of bubble and decreasing its size favors gas transfer and enhances the bioprocesses. Microorganisms and bubbles are known to exchange gas due to collisions and interfacial interactions bringing the two phases in close proximity (Gilmour and Zimmerman 2020). Consequently, prolonged transport followed by sustained oxygen transfer plays a pivotal role in microbial and fermentation systems. Similarly, supplementation of media with NB water increased the mycelium concentration as well as the yield of polysaccharides while also enhancing mycelial bioactivity (Xiao et al. 2020). Owing to high density, mycelial filaments impede mass transfer. NB water, owing to its high spin‐spin relaxation time (*T*_2_), increases mobility of water molecules, and the zeta potential creates repulsion preventing coalescence, together favoring biomass accumulation (D. Wang, Yang, et al. 2019). A recent study from bacterial nanocellulose production demonstrated a nearly six‐fold higher kLa and oxygen transmission rate mediated by NBs relative to a conventional airlift reactor at the same airflow rate. This supports the role of NBs in increasing gas–liquid interfacial area and oxygen residence. However, despite increased oxygen availability, only cell density and substrate consumption increased, whereas the production of bacterial nanocellulose was still comparable to conventional aeration, indicating that oxygen delivery by NBs needs to be optimized for higher production of metabolites rather than simple maximization (Cristina et al. 2026). NB‐mediated oxygen transport additionally promoted the growth of microalgae, like *Haematococcus lacustris* and *Botryococcus braunii*, known for the production of astaxanthin and lipids, respectively, principally attributed to an increase in biomass (Zhu and Wakisaka 2019). Chlorophyll and carotenoid contents also increased in NB water supplementation to the media growing *Nannochloropsis oculata* and *Chlorella vulgaris* (Choi et al. 2014). Sustained dissolved oxygen availability also improved the growth and food consumption of rainbow trout and sweet‐fish in NB water systems (Ebina et al. 2013). These findings collectively indicate that prolonged gas transport directly influences diverse biological systems ranging from microbial fermentation to aquatic growth and anaerobic bioprocesses. NBs were also investigated for their influence on biomethane and biohydrogen production during anaerobic digestion, where positive effects on bacterial growth and enzyme activities enhanced gas production efficiency (Patel et al. 2021; D. Wang, Yang, et al. 2019). Although the sustained residence of oxygen NBs generally enhance the availability of oxygen, nitrogen NBs are seen to play an antagonistic role by displacing dissolved oxygen from the system. N_2_ NBs reduce dissolved oxygen and lower oxidation–reduction potential thereby creating a low‐oxygen aqueous environment suitable for delaying aerobic spoilage reactions (K. Zhang, Feng, et al. 2026). This implies gas transport can have varying outcomes depending on the type of gas used in the system.

Beyond microbial and fermentation systems, prolonged gas retention also influences the colloidal and foam stability in food systems. Incorporation of NBs maintained the foam structure in instant coffee. The small size of NBs, coupled with high stability, retains gas in the liquid and increases its solubility, which further slows down its transport at the air–liquid interface, and thus, a foam structure is kept intact (Deotale et al. 2020). This highlights the functionality of NBs mediated by sustained transport that is regulated by underlying electrokinetic mechanisms, rather than gas dissolution alone.

### 5.3 Interfacial Micro‐Convection and Transport Amplification in Food Systems

NBs significantly enhance transport processes by inducing localized turbulence and fluid flows, nano‐jets, and shock‐waves produced by collapse at the gas–liquid interface. The localized microflows generated during bubble collapse thin the diffusion layer thereby reducing resistance surrounding solid matrices, which enhances solvent accessibility and diffusion of intracellular compounds. These interfacial transport phenomena become particularly important during extraction of phenolics, flavonoids, and other bioactive compounds from plant tissues, by forming permanent nanopores and physically disrupting cell walls and membranes, improving solvent penetration into plant material and the release of intracellular compounds (Figure 5) (Ettoumi et al. 2022). Additionally, the high surface‐area‐to‐volume ratio of NBs promotes efficient contact between solvent and plant tissues, facilitating mass transfer during extraction processes (Javed, Belwal, Huang, et al. 2022).

CO_2_ NBs generated through compression–decompression method and stabilized using surfactants followed by ultrasonication significantly improved phenolic compound recovery from *Camellia oleifera* shells compared to conventional extraction methods. Rhamnolipid and Tween‐80 were used as surfactants that reduced surface tension and increased negative zeta potential of the NBs, conferring stability against coalescence. Consequently, more interfacial reactions were favored as a result of stable NBs. A higher total phenolic content of 34.42 mg gallic acid equivalent per gram dry weight (mg GAE/g DW) and flavonoid content of 23.36 mg rutin equivalent per gram dry weight (RE/g DW) were reported (Javed, Belwal, Ruyuan, et al. 2022). Another study additionally optimized polyphenol extraction from peanut shells using a CO_2_ NB‐assisted ultrasound extraction method. The CO_2_ NBs were produced in distilled water using a custom high‐pressure nano‐jet homogenizer, showcasing the potential of this advanced approach to revolutionize extraction processes. The combined treatment yielded significantly higher polyphenol content compared with ultrasound treatment alone. Due to high specific surface area and high stability of nano bubbles in liquid, they accumulate at the tissue surfaces, pores, and microcracks in plant tissues. During ultrasonic treatment the bubbles collapse, generating intense localized shear forces and turbulence. The cavitation effects are enhanced by CO_2_ NBs acting as nucleation sites, leading to stronger and more uniform bubble collapse (Iqbal et al. 2025). This needs to be differentiated from intrinsic NB collapse as it is mediated by ultrasound waves. The NBs here act as preexisting gas nuclei, the existence of which is also due to intrinsic effects, such as long residence time, high interfacial area, and gas delivery, to be activated into cavitation phenomena by ultrasound. As a result, plant cells damage is pronounced, diffusing out the intracellular polyphenols more rapidly into the solvent. In addition, dissolved CO_2_ can slightly lower the pH of the extraction medium through carbonic acid formation, which may improve the solubility and stability of certain phenolic compounds (Iqbal et al. 2025). These outcomes are therefore coupled static‐NB‐ultrasound cavitation induced for the studies under consideration. As the polyphenol content was higher when ultrasound was combined with NBs, it indicated that NBs multiply the cavitation effects. Likewise, nitrogen NBs in ionic liquids (IL)∖[C4C1im]∖[BF4] were used to extract polyphenols from *Carya cathayensis Sarg*. The NBs were generated using compression–decompression and then ultrasound was applied for inducing cavitation. From the results, it was evident that IL‐NB extraction exhibited superior antioxidant potential measured by total phenolic and flavonoid content, as compared with ethanol and IL alone. This is due to the fact that increasing IL concentration decreased the size of NB along with increasing its zeta potential, thus increasing stability. In water, a negative surface potential exists on the NB surface due to abundance to OH^−^ ions at bubble surface. On addition of IL, a charge reversal from negative to positive zeta potential was seen, as the nonpolar tail of [C_4_C_1_im]^+^ attaches to gas–liquid interface, positioning itself towards the gas, thereby conferring positive charge to the bubble surface towards the bulk. ILs can strongly disrupt plant cell walls and improve the release of bound phenolics due to their unique ionic interactions. In this study, however, IL extraction alone could not bring comparable results relative to when used with NBs, highlighting the positive effect of NBs, which was also confirmed by microscopic studies showing more pores, cell wall destruction, and roughness that enabled improved transfer (Ettoumi et al. 2022; Ma et al. 2022; Moreno et al. 2008). The efficiency of phenolic extraction employing NBs depends on combined control of solvent properties, stability of NB, gas pressure, bubble size and concentration, and cavitation parameters. NBs that are small, stable, and concentrated can provide more nuclei for cavitation and a longer contact with matrix (Iqbal et al. 2025; Javed, Belwal, Ruyuan, et al. 2022), whereas the properties of solvent, like polarity, acidity, viscosity, solvent‐phenolic interactions, and gas solubility, will determine phenolic solubilization, residence of bubble, and penetration of solvent in the matrix (Bajkacz et al. 2020). Similarly, the ultrasonic energy applied should be sufficient to induce cavitation without having deleterious effects on the phenolics. For instance in the study of Ettoumi et al. (2022), increasing IL concentration from 1 to 2 M reduced NB size from 85 to 41 nm, compared to 246 nm in water. Increasing nitrogen pressure reduced NB size from 102.6 to 70 nm, along with increasing zeta potential. NB concentration decreased with increasing ultrasonic energy implying their collapse, appearing as decrease in measurable number. However, beyond 300 W, application of additional energy could not produce any collapse implying attainment of optimum threshold, beyond which the phenolics could be exposed to risk of degradation. Conversely, unstable NBs, improper solvent selection for phenolic dissolution, insufficient interfacial contact due to lesser number of NBs and degradative external cavitation will negatively affect polyphenol extraction. This negative effect was evident in the study of Ai et al. (2024), where ultrasound cavitation caused NB rupture, along with damaging plant surface and extraction enzyme, thereby reducing extraction efficiency. CO_2_ NBs were generated by compression–decompression and combined with cellulase enzyme to extract phenols from *Polygonatum cyrtonema* compared with conventional enzymatic hydrolysis and without ultrasound. Cellulase resulted in smaller NBs and increased their number acting like a surfactant that promoted bubble formation, consequently improving their dispersion and interaction with plant matrix. The long residence of NBs in the system was also due to their intrinsic negative charge and electrokinetic effects. The NBs aggregated on the surface of plant matrix added as powder into the system, leading to an increased local pressure that caused intrinsic cavitation of NBs and lead to loosening of plant matrix, creation of pores, and fragmentation. This finally led to improved solvent and enzyme penetration followed by phenolic diffusion. Combined CO_2_ NBs and cellulase produced higher extraction than using cellulase alone and was evident through notable microscopic fragmentation of the plant matrix. Use of ultrasonic waves produced the least yield owing to destabilization of NB‐enzyme system highlighting the need to optimize cavitation intensity.

These interfacial transport amplification principles are additionally relevant during membrane cleaning and antifouling applications. As NBs generate localized turbulence and micro‐convective flow, it enables the disruption of fouling layers and reducing diffusional limitations near membrane surfaces. Due to these attributes, NBs were used for the removal of proteins adsorbed on solid surfaces. The percentage of removed protein increased by more than 7 on incorporation of NBs in cleaning water (H. Chen et al. 2009). Similarly, the removal of biofilms and soiling mixtures, such as carbohydrates, proteins, or fats, from stainless steel and polypropylene surfaces was achieved through the administration of air bubbles in water. The combinatory treatment enabled effective cleaning with less water and doubled reduction of microorganisms compared to normal water (T. Phan et al. 2020; Burfoot et al. 2017). NB incorporation in the filtration membranes additionally prevented membrane fouling owing to enhanced transfer capacity (Zhu et al. 2016). Due to electrostatic repulsion, NBs introduce turbulence in the bulk liquid, promoting agitation, consequently removing particles from the membrane surfaces (Foudas et al. 2023). Kobayashi et al. (2025) showed that air, O_2_, CO_2_, and N_2_ NB waters were effective in removing biofilms produced by *Escherichia coli* relative to deionized water, with removal efficiency following N_2_ > CO_2_ > O_2_ ≈ air. N_2_ NBs exhibited a reduced surface tension that enabled penetration into biofilm layer and favored the detachment by biofilm stripping and hydrophobic interactions at the biofilm–bubble interface, highlighting the dependence of biofilm removal on gas‐type. In contrast, O_2_ NBs increased the concentration of dissolved oxygen and may even favor biofilm formation as a result. For CO_2_ NBs, the removal efficiency was because of acidic pH that can weaken the biofilm matrix followed by stripping. This distinguishes physical mechanism of biofilm removal from O_3_ NBs‐mediated microbial killing involving chemical entities. These studies show that NBs can be integrated into strategies to ensuring hygiene for equipment surfaces, packaging lines, and fresh produce handling.

Likewise, in dairy‐processing systems NBs are incorporated into milk, enabling the increase of flux and reduction of ultrafiltration time. Membrane deposits are significantly reduced for NBs‐incorporated skim milk concentrate dispersions due to increased rate of flux, reducing the energy required for pumping. This is because NBs create turbulence, reduce viscosity of fluid, and cause breaking of fouled layer (Babu and Amamcharla 2023a).

The turbulence that comes into effect as a result of NB collapse also influences colloidal aggregation behavior in protein‐rich food systems. The incorporation of NBs in Greek‐style yoghurt supplemented with micellar casein improved its rheological and functional attributes. NBs led to disruption of aggregates of casein and consequent reduction in viscosity, as well as reduction in syneresis during storage (Babu et al. 2022). Similarly, microscopic analysis of milk protein concentrate dispersions treated with NBs revealed reduced aggregation and more pronounced alterations in structure compared to conventional dispersions, significantly reducing the viscosity (Babu and Amamcharla 2022). Likewise, in a sweetened fermented dairy drink supplemented with whey protein concentrate, compressed‐air micro‐nano‐bubbles reduced syneresis and prevented protein aggregation thereby reducing apparent viscosity. The water‐holding and flow properties were improved, which produced smoother textures and more homogeneous dispersion conferring structural stability to the fermented dairy drink (Kaura et al. 2026). The conversion of large protein aggregates into less dense structures is due to the breakdown of NBs, which sends shockwaves and generates turbulence through the medium that causes disruption of protein aggregates.

These findings suggest that enhanced extraction of bioactives from plant matrices, antifouling properties, and hydrodynamic membrane cleaning may originate from the same interfacial transport mechanisms involving turbulence induced by collapse of bubbles, enhancement of diffusion, and electrokinetically regulated local micro‐convection around NB interfaces.

### 5.4 NBs‐Mediated Matrix Modulation and Food Structure Control

Food systems constitute a complex colloidal environment of proteins, polysaccharides, lipids, salts, water molecules, and dispersed particles capable of interacting strongly with NB interfaces. Interactions with these macromolecules can substantially modify interfacial charge distribution, electrokinetic, and colloidal behavior, thereby influencing structural and rheological functionality within food matrices. Food components, like proteins and polysaccharides, can change the permittivity of the medium as well as the zeta potential that can, in turn, modify the diffusion layer. Similarly, proteins and polysaccharides also adsorb at the NB interface thereby modifying the surface charge and electric double layer (Figure 5). As transport persistence and colloidal mobility strongly depend upon interfacial electrokinetic behavior, these adsorption phenomena can govern food matrix restructuring. Proteins, such as casein, when adsorbed at the gas–liquid interface may induce charge neutralization and hence destabilization. Protein can induce immobilization of bubble surface due to getting irreversibly adsorbed; however, remobilization of the bubbles stems from interfacial properties particularly at high concentrations (Witkowski et al. 2025). Moreover, structural deformation of protein may come into effect on adsorption at a static gas–liquid interface depending on protein net charge and structure (Gochev et al. 2024). In contrast, pectin adsorption, however, can enhance electrosteric balance providing prolonged lifetime to NBs and hence increased mass transfer, as it is negatively charged (Zhang et al. 2020). Such electrosteric interactions can contribute to improved colloidal stability and restructuring within food matrices.

NBs influence crystallization behavior within dairy systems through interfacial restructuring and transport modulation. NBs act as catalysts, increase nucleation sites, and alter crystallization reactions resulting in the formation of smaller, more uniformly distributed lactose crystals, while reducing the need for added seeding material. Research studies have demonstrated that incorporating CO_2_ and N_2_ micro‐NBs increased the lactose crystal yield compared to untreated systems (Adhikari et al. 2020). Similarly, the incorporation of NBs into anhydrous milk fat has been shown to enhance fat crystallization by facilitating the formation of numerous smaller sized crystals during the cooling process (Xun et al. 2017). The presence of CO_2_ micro‐NBs altered the crystallization behavior by favoring an earlier onset of crystallization and accelerating overall crystallization rate in anhydrous milk fat systems (Adhikari et al. 2020). Apart from modulating crystallization behavior, NBs also improve structural properties of dairy systems by acting as buffering agents at the interface. Due to the charge at the gas–liquid interface, NBs act as physical and electrostatic buffers between proteins limiting their interaction and consequent aggregation and clumping. Interactions between NBs and hydrophobic regions of casein produce a structurally dispersed protein network with lower viscosity. NBs also reduce intermolecular attraction among water molecules and redistribute them in the gel network ensuring lower viscosity and improved water‐holding capacity, thereby reducing syneresis during storage (Akshit et al. 2024).

NBs are also known to regulate freezing kinetics based on the type of gas used. Air NBs were seen to delay water solidification by up to 8.7% ± 1.9%, whereas CO_2_ NBs accelerated it by up to 11.4% ± 2.5%, without any observed freezing‐point depression. Air NBs were proposed to delay freezing because negatively charged OH^−^‐rich interfaces are expelled toward unfrozen regions during ice growth, where they may disturb ordered ice‐lattice formation, deplete protons, and physically impede ice‐front progression. In contrast, CO_2_ NBs accelerated solidification because CO_2_ dissolution produced bicarbonate/carbonate species that favor ice embryo formation and promote ice growth. This suggests that NBs should be selected on the basis of the processing goal, rather than treating all NBs as nucleation sites in frozen foods. CO_2_ NBs may be used for faster freezing applications to ensure product quality, whereas air NBs may be relevant where delayed ice growth or prevention of freezing is useful (Yamamoto and Kioka 2026).

NB‐mediated restructuring also influences foam stability and textural behavior in frozen food systems. Ice cream mixes prepared using NB water demonstrated lower overrun compared to control samples, primarily due to the presence of ultrafine air bubbles that occupied less volume despite their higher density, unlike the larger air cells in conventional ice cream. The ice cream supplemented with NBs retained its body longer than the conventionally prepared ice cream as it had high firmness and dense texture (Khaira et al. 2020). The nanoscale dimensions of NBs altered air‐cell distribution and interfacial organization within the frozen matrix. Smaller bubbles created thin lamellae between air cells that reduced liquid drainage along with acting as insulators, preventing heat transfer and consequently slowing melting. Conversely, incorporation of CO_2_ NBs into soft ice cream resulted in a significantly higher overrun (88%) compared to the control (26%) (Adhikari et al. 2020). This behavior may arise due to pre‐seeding of ice cream mixture with CO_2_ NBs in comparison to air incorporation during churning, that is more uniformly distributed by agitation during churning and remains entrapped in the structure.

NBs were also employed in fabrication of porous cryogels on the basis of chitosan and silk fibroin. Incorporation of NBs into solutions before producing cryogels interfered with network organization during freezing, resulting in branched pore structures of smaller sizes and altered internal structure. NB neutralized the positive charges within pre‐solutions and enhanced hydrogen bonding between macromolecular chains, increasing pore expansion and structural organization. These pores remarkably increase oil absorption capacity, which can enable the production of oleogels and their application in the food industry (Q. Jiang et al. 2025).

NBs may also regulate crystallization in food matrices by providing charged gas–liquid interfaces for heterogeneous nucleation. During cooling crystallization of glycine, O_2_ and N_2_ NBs shortened the induction time, increased yield, and reduced crystal size, with O_2_ NBs producing smaller crystals than N_2_ NBs. In comparison to ultrasound, NBs exhibited comparable crystal‐size reduction with the expense of lower energy input and less risk of thermal degradation. This can have relevance for food systems such as sugars, salts, fats, and frozen matrices that are relevant for crystallization. However, increasing the flow increased crystal size, highlighting the need to optimize bubble density in the system rather than maximum bubble generation. Also, O_2_ NBs were found to produce smaller sized crystals due to higher solubility in water that may provide more effective gas–liquid interfacial sites for heterogeneous nucleation (A. Sharma et al. 2026).

Similar restructuring behavior has additionally been observed in lipid and powder systems. NBs reduced the hardness of palm oil, by favoring crystallization and formation of uniform fat crystals during cooling. It was because the crystal network of fat in palm oil was disrupted by CO_2_ NBs, reducing hardness compared to untreated oil systems (Phan et al. 2024). Likewise, incorporation of N_2_ NBs improved properties of spray‐dried maltodextrin powder by reducing the viscosity, altering powder morphology, promoting spherical particle shape, and decreasing moisture content (Oh et al. 2024). Collectively, these findings indicate that adsorption‐mediated restructuring at the interface governs crystallization, foam network, pore architecture, and rheological modification within complex food matrices.

### 5.5 Reactive Species Transport and Redox Functionality

NBs possess the capability to generate ROS, including hydroxyl radicals, superoxide radicals, singlet oxygen, and hydrogen peroxide owing to elevated localized pressure and temperature generated during bubble collapse (Figure 5) (Javed et al. 2023). The internal pressure of bulk NBs for a liquid temperature of 298 K is higher than that of the external pressure by about 2.9 times. On the collapse of NBs, the internal pressure tending to be infinite leads to a temperature increase to 5000 K at that point, disrupting water vapor and gases inside bubbles producing free radicals, the concentration and size of which vary by their size and gas employed (Takahashi et al. 2007). However, the functionality of NBs in food preservation and microbial decontamination is governed not merely by ROS generation, but by sustained transport and localized delivery of reactive species facilitated through electrokinetic stabilization and prolonged transport persistence. Electrokinetic stabilization prolongs NB persistence and thereby enables sustained ROS that are instrumental in oxidative interactions with microbial cells and food surfaces. Simultaneously, the nanoscale dimensions of NBs facilitate penetration into microbial biofilms and surface irregularities, enabling localized oxidative interactions. ROS transported by NBs interact with microbial membranes causing lipid peroxidation, membrane destabilization, leakage of intracellular contents, and eventual microbial inactivation (Ushida et al. 2017). This apparent contrast with microbial growth promotion discussed in Section 5.2 is not contradictory, but it reflects interactions of NB with microbes based on the gas type and condition. In systems with dominant gas persistence, enhanced microbial growth and metabolite production will be observed, whereas NBs containing oxidative or reactive gases will intensify microbial inactivation. Studies based on modeling these interactions have indicated that NB‐assisted inactivation may involve both spontaneous inactivation and ROS‐mediated reduction, particularly for CO_2_, N_2_, O_2_, and ozone‐containing systems (Ulatowski et al. 2026). For instance, the prolonged residence time and transport persistence of ozone NBs particularly enhance oxidative delivery within food systems. Ozone dissolved within NBs exhibits slower dissipation compared with conventional ozonated water, thereby prolonging antimicrobial activity and oxidative transport. These are particularly important in ozone‐assisted food preservation systems, which exhibit prolonged oxidative ability as a result of sustained interfacial stability, enabling localized transport of ROS towards microbial surfaces. Such sustained transport has been associated with reduced microbial load in chestnuts and Chinese cabbage (U. Lee et al. 2016; Ushida et al. 2017).

NB technology, when combined with different gases, such as CO_2_, O_3_, and C_2_H_4_, has demonstrated significant impacts on the postharvest quality and shelf‐life of various food and agro‐products (Kumari et al. 2018; Malahlela et al. 2024). Ozone NB treatment for a period of 3 min in Chinese cabbage led to a reduction in bacterial counts and delayed quality deterioration (Ushida et al. 2017). Similarly, a 5‐min O_3_ NB treatment followed by storage at 20°C for 8 days resulted in improved sensory qualities and antioxidant enzyme activities while reducing oxidative deterioration and respiration‐induced quality loss in spinach, ultimately extending the shelf life (X. Wang et al. 2020). In chestnuts, a 10‐min O_3_ NB treatment followed by storage at ∼4°C and 90% relative humidity for 16 weeks improved sensory attributes and decreased aerobic bacteria, molds, and yeasts, contributing to delayed quality degradation (Lee et al. 2016).

NBs can also function as washing media for reducing microbial contamination on fresh produce. Malahlela et al. (2025) compared gas‐dependent antimicrobial efficacy of micro‐nano‐bubble waters against *E. coli* and *Staphylococcus aureus* in bacterial suspensions and on inoculated guava fruit surfaces. Although air‐micro‐nano‐bubbles had the smallest bubbles and highest concentration, their low oxidation–reduction potential resulted in limited microbial inactivation, because the air is chemically mild. O_2_ micro‐nano bubbles were seen to cause moderate bacterial injury after longer exposure, whereas O_3_ micro‐nano bubbles produced the strongest effect due to low pH, high oxidation–reduction potential, and ozone‐derived reactive species. On microscopic confirmation, various changes in the microbial cells were observed such as membrane injury, detachment of the cytoplasmic membrane from the cell wall, loss of intracellular/DNA structures, leakage of cellular contents, deformation of cell morphology, and eventual lysis. This confirms oxidative and structural destruction of microbial cells through ozone‐derived reactive species, rather than simple microbial detachment from the fruit surface (Malahlela et al. 2025). Further, it was observed in pork that the delivery mode strongly improves ozone NB efficacy when it is shifted from simple immersion to a dynamic flow rinse. This is attributed to renewed contact of ozone with the NB at the pork surface and limited depletion of ozone by organic matter. This indicates that ozone‐NB sanitation in meat systems depends not only on ozone concentration but also on solution flow rate and dynamics of surface‐contact. The study also showed that O_3_ NBs altered pork microbiota by reducing spoilage‐associated microbes, such as *Vagococcus* and *Clostridium*, suggesting potential relevance for shelf‐life extension and blown‐pack spoilage control (Z. S. Xu et al. 2025).

O_2_ NBs have been combined with edible coatings to improve refrigerated shelf life in seafood. Chitosan + O_2_ NB treatment was more effective than either component alone in maintaining shrimp quality, as reflected by lower microbial counts, and reduced oxidation parameters, and better texture and sensory acceptability. Chitosan provides an oxygen barrier and electrostatic antimicrobial action, whereas O_2_ NBs may improve interfacial contact and contribute to microbial stress. However, because O_2_ is also known to promote lipid oxidation, the benefit of O_2_ NBs in seafood depends upon formulation and may require protective coatings such as chitosan to prevent oxidative quality loss (Kanani et al. 2026).

In aquaculture systems as well, the disinfection efficacy of NBs was observed by Thi et al. (2025) as a function of dose and matrix. O_3_ NBs treatment was found to suppress the growth of *Saprolegnia* and eliminated visible *Saprolegnia*, *Dermocystidium*, and *Fragilaria* infections from common carp embryos, but subjecting the larvae to direct exposure caused severe gill damage and mortality even at lower ozone concentrations. This contrast shows that the effectiveness of O_3_ NB in microbial control is not governed only by efficient oxidation but also by tolerance capacity of the tissue, exposure stage, and treatment duration. This study can have direct implications for food sanitation as disinfection is governed by a balance between oxidative antimicrobial efficacy and stability of matrix post exposure. Similar optimization of ozone concentration, exposure time, and gas type is required for fresh produce, seafood, or minimally processed foods, to achieve microbial reduction without inducing quality loss and sensory deterioration as a result of oxidation (Botondi et al. 2021; L. Wang, Fan, et al. 2019). Therefore, food applications require a balance between microbial reduction and preservation of tissue integrity, sensory quality, and nutritional stability.

O_3_ micro‐NBs are also relevant for chemical decontamination, particularly pesticide residue reduction on fruit and vegetable surfaces. Their effectiveness is mainly linked to persistent oxidative delivery allowing ozone‐derived reactive species to interact more persistently with surface contaminants. The process, however, depends on residue chemistry, surface characteristics of produce, bubble generation method, ozone dose, and treatment time (Pal and Kioka 2025). In a study carried out by X. Li et al. (2026), combining air micro‐NBs with ultrasound was seen to enhance the oxidative capacity of water in comparison to either treatment alone. An improvement in redox behavior was associated with small size of the bubble, higher dissolved oxygen, lower pH and cavitation‐assisted generation of oxidative species. To increase air incorporation and ensure high interfacial area, high‐speed vortexing was adopted, whereas ultrasound promoted cavitation leading to the diffusion of oxidative species into liquid phase for pesticide degradation. The study indicated that NBs support reactive species generation and transport when synergized with an external cavitation influence.

While ozone and oxygen‐containing NB systems predominantly promote oxidative interactions for microbial inactivation and decontamination, hydrogen‐rich NB are seen to exhibit contrasting behavior by mitigating oxidative stress within food matrices (Figure 5) (H. Chen et al. 2017). Liu et al. (2018) showed that hydrogen NB water scavenged •OH, ClO^−^, ONOO^−^, and O_2_•^−^, while showing limited activity against H_2_O_2_ and NO, indicating selective ROS regulation instead of broad oxidative disinfection. Consequently, the functionality of NBs in food systems depends not only on transport persistence, but also on the physicochemical nature of gas and its associated redox activity. Hydrogen‐rich NB systems have demonstrated significant antioxidant‐preserving effects during food storage. Treatment with hydrogen‐rich water improved antioxidant activity, nutritional quality, and oxidative stability in mushrooms, litchi fruit, Chinese water chestnuts, and *Rosa sterilis* during storage. Across the studies, reduction of H_2_O_2_, increase in antioxidant enzyme activity, reduction in electrolyte leakage, and quality loss were observed (H. Chen et al. 2017; Dong et al. 2022; Lee et al. 2016; Yun et al. 2021). These improvements were directly associated with reduced oxidative stress and enhanced antioxidant defense, indicating the capability of NB‐mediated transport to regulate oxidative pathways within food matrices.

However, NBs always do not exhibit positive outcomes as was the case with fermented milks. The redox functionality of gases, such as CO_2_ and N_2_ + H_2_, was examined in fermented milks where it was hypothesized that their administration would reduce the oxidation–reduction potential and therefore improve longevity of probiotics. Among the strains tested, only *Lactobacillus rhamnosus* populations remained higher in NB supplemented milk than the control. However, for milk fermented with this strain there was no significant effect of NBs on oxidation–reduction potential and dissolved oxygen. As the pH of milk was 5, which is not low enough to inhibit probiotics, no significant beneficial effects of NBs were concluded. Therefore, the probiotic survivability was not attributed to redox activity warranting more studies for better understanding (S. Sharma et al. 2023).

In addition to ROS generation, sanitation of fresh‐cut produce may also combine NB‐induced acidic, oxidative, and mechanical stresses. In fresh‐cut lotus root, CO_2_ micro‐NBs combined with H_2_O_2_ and intermittent ultrasound achieved the highest *E. coli* O157:H7 reduction, with its efficacy being associated with changes in the membrane permeability, ultrasound–bubble interactions, CO_2_‐related acidification, and H_2_O_2_‐mediated oxidation, rather than a sole ROS‐driven process. Air micro‐NBs + H_2_O_2_, however, were more strongly associated with intracellular oxidative stress (Shi et al. 2026).

Similarly, nitrogen NBs provide preservation route of non‐oxidative nature based on oxygen displacement and redox suppression. N_2_ NBs were prepared by mechanical shearing along with controlling gas flow, pressure and shearing time. With an increase in shearing time and gas flow, bubble density and pH slightly increased. Longer shearing time decreased ORP as more NBs were generated to displace oxygen. The resulting N_2_ NBs exhibited delayed deterioration in spinach, apple, and pork during room‐temperature storage. The preservative effect was associated with reduced dissolved oxygen, lower ORP, inhibition of aerobic microbial metabolism, and delayed oxidation‐driven spoilage (K. Zhang, Feng, et al. 2026). These comparative insights imply that NBs‐mediated preservation is gas‐specific, such that O_3_ NBs act mainly through oxidative stress and membrane damage, H_2_ NBs operate through antioxidative protection, and N_2_ NBs through oxygen exclusion, ORP reduction, and suppression of aerobic spoilage pathways.

Oxidative transport mechanisms are additionally relevant in membrane cleaning and antifouling systems. On contact surfaces, NBs precipitate the salt by attracting counterions on their surface and introduce hydroxyl radicals that degrade contaminating entities (Foudas et al. 2023). Application of pilot and bench scale ceramic membrane in combination with 100 nm air NBs was found to prevent fouling as well as offer cleaning by ROS generated as a result of bubble collapse (Ghadimkhani et al. 2016). Ozone NB water was also evaluated for multispecies biofilm control in pork‐processing environments, the activity being based on oxidative damage mediated by surface contact and penetration into biofilm matrix. The results revealed that the reduction in biofilm was modest and defined by the target community indicating the dependence on the nature of biofilm, exposure, and operating conditions (Y. C. Jiang and Gänzle 2026).

Among plants also, NBs promote growth by favoring seed sprouting that can address a rising concern of the rapidly increasing human population. In this context, the effect of different gas NBs on germination and growth of various seeds, like lettuce, carrot, fava bean, and tomato, was examined. Under the administration of O_2_, N_2_, and CO_2_ water, seed germination increased by about 6%–25% as compared to that with tap water. This is primarily because of the ROS generated by NBs that cause cell wall elongation and loosening, thereby promoting growth (Ahmed et al. 2018).

Therefore, oxidative decontamination, shelf‐life extension, membrane cleaning, and oxidative antifouling functionality all emerge from sustained reactive species transport and electrokinetically regulated oxidative interactions occurring around NB interfaces.

### 5.6 Transport Persistence and Bioactive Delivery Systems

NBs are being explored as interfaces for encapsulation, stabilization, and controlled delivery of bioactive compounds within food systems. The sustained interfacial persistence and colloidal stability of NBs can favor controlled dispersion and regulated release of nutraceutical compounds (Figure 5). Electrokinetic stabilization contributes significantly to this functionality by suppressing aggregation. Consequently, NB‐assisted delivery systems may exhibit prolonged stability and interfacial contact, which are important for encapsulation and release behavior. Investigating this domain, functional hydrogels mediated by the addition of CO_2_ NBs were fabricated for the delivery of gallic acid, that effectively encapsulated and supported its controlled release. The addition of NBs improved the rheological and mechanical properties and also promoted the formation of a compact network with improved encapsulation and sustained‐release behavior (Javed et al. 2024). Similarly, Temprom et al. (2025) demonstrated that a noisome‐NB based colloidal system enhanced encapsulation efficiency and sustained release of lycopene owing to improved colloidal stability and prolonged persistence. Compared with other food applications of NBs, their use in encapsulation and controlled‐release systems remains comparatively emerging. However, recent studies indicate that NBs can act as transport‐mediating interfaces capable of improving dispersion stability, encapsulation efficiency, and sustained release of bioactive compounds.

## 6 Limitations and Future Scope

Although NB technology displayed a great promise for applications in the food industry, active research and development efforts remain limited. Despite promising attributes, NB application faces certain constrictions such as gas‐specificity implying that their usage cannot be generalized across food systems. The varying outcomes of same NBs warrant for more deeper studies to establish primary application field. Current evidence indicates that the effects of NBs on microorganisms are highly dependent on the gas, size, concentration, and condition of operation, underpinning need for future studies that can distinguish the effect of stable NBs from those of cavitation‐induced dynamic effects and also lay foundations for scale‐up (Ulatowski et al. 2026). For other applications, involving cavitation induced effects, the observed outcomes cannot be attributed solely to NBs or ultrasound, and therefore, the current literature does not distinguish stable and dynamic effects, calling for future studies to report ultrasound‐only and NB‐only controls in addition to conventional controls without either or combined treatment. Conversely, ultrasound can have negative implications in certain cases where they can rupture stable NBs, increase bubble size, reduce the concentration of NBs, enzyme or matrix disruption as was evident in enzyme‐assisted NB extraction and therefore reduce extraction yield (Ai et al. 2024). The antimicrobial action of O_3_ NBs is often nonselective and warrants more experimentation. O_3_ NBs promote radical mediated oxidation and microbial inactivation, it can also damage tissues, lipids, pigments, nutrients, or beneficial microflora if the exposure conditions are not optimized. It was also observed that improved gas transfer doesn't always necessarily translate into a desired outcome, for instance increase in microbial biomass due to gas exposure doesn't lead to increased metabolite production (W. Wang et al. 2026). Antioxidant NBs are seen to bring beneficial effects in preservation and decontamination; however, these should not be assumed to be universally beneficial, because excessive ROS scavenging may disturb normal physiological processes as evidenced by hydrogen NB water inhibiting barley seed germination by eliminating endogenous O_2_•^−^ required for germination (Liu et al. 2018). Organic‐rich food matrices, such as meat, can reduce ozone efficacy, so optimization of ozone concentration, liquid volume, flow rate, and contact time is essential before industrial application (Z. S. Xu et al. 2025). Similarly, some microbial communities resist biofilm removal on the application of ozone, limiting its established role as a sanitizer (Y. C. Jiang and Gänzle 2026). There is still limited standardization in the generation, characterization, and reporting under real food‐processing conditions and therefore future applications should aim outcome‐targeted, gas‐defined and matrix‐oriented applications in NBs in food systems. There is also a need for standardization of bubble generation, control of bubble size/concentration, energy efficiency, scalability, and long‐term validation (Balea et al. 2026).

Nevertheless, the unique attributes of NBs such as their ability to enhance quality and ease of generation, make them a strong candidate for future food applications. The use of NBs to improve gas–liquid phase interactions offers significant potential across various food processes. Future studies should take into account the complex nature of food matrices and the NBs action. Specifically, investigations should explore how NBs treatment parameters, such as gas type, bubble size, exposure duration, and commodity type, can influence nutritional and bioactive compound content in fresh fruits and vegetables, dairy industry, and extraction process. Additionally, it is important to examine how NBs affect physiological processes such as metabolic activity, respiration rate, and the delay of natural senescence during storage (Malahlela et al. 2024). The effect of NB application on food constituents, its sensory appeal, and overall quality as defined by different types of NBs needs to be looked into. In processes involving reactive species, it is essential to thoroughly evaluate the dynamics of reactive species involved, the chemical reactions they undergo, and their migration within the food matrix, to prevent the formation of toxic compounds. Moreover, a wider framework of food applications of NBs, their commercial viability and applicability, economics involved and sustainability, and also the effect on food materials yet remain unexplored.

## 7 Conclusions

NB technology represents an emerging green and sustainable approach, utilizing low‐cost gases, which contributes to its broad potential for future applications across various fields. NBs have demonstrated the ability to enhance the extraction of phytochemicals, improve solubility, promote nucleation and crystallization processes, minimize membrane fouling, and boost the effectiveness of antimicrobial agents principally governed by electrokinetic stabilization and sustained transport leading to efficient interfacial interactions. The basis of functionality has been reviewed as supported by electrokinetic stabilization and sustained transport, and further discussed as specific mechanisms leading to food outcomes. In food systems, the NB functionality is based on distinct mechanisms be it sustained transport, hydrodynamic interactions, matrix modulation, and redox functionality. The varying functionality is also defined by gas‐properties, process conditions, and matrix interactions. Future research should focus on integrating the chemical, physical, and hydrodynamic properties involved in NBs generation to develop energy‐efficient, technically robust, and user‐friendly methods for producing bubbles with controllable sizes. Moreover, industrial‐scale research focusing on reactor design, energy efficiency, and process optimization should be explored. Overall, the application of NBs in food sciences holds considerable promise; however, to fully realize their potential, further research is essential to understand the fundamental behavior of NBs, their interactions within complex food matrices, and to enable consistent, large‐scale production for commercial food processing.

## Author Contributions

**Farah Naqash**: writing – original draft, visualization, conceptualization, writing – review and editing. **Miral Javed**: writing – original draft, visualization, conceptualization, writing – review and editing. **Akmal Nazir**: writing – review and editing. **Sajid Maqsood**: visualization, supervision, conceptualization, writing – review and editing, funding acquisition.

## Conflicts of Interest

The authors declare no conflicts of interest.
